# CAR T-Cells in Acute Lymphoblastic Leukemia: Current Status and Future Prospects

**DOI:** 10.3390/biomedicines11102693

**Published:** 2023-10-02

**Authors:** Abdulrahman H. Almaeen, Mohamed Abouelkheir

**Affiliations:** 1 Department of Pathology, Pathology Division, College of Medicine, Jouf University, Sakaka 72388, Saudi Arabia; ahalmaeen@ju.edu.sa; 2 Department of Pharmacology and Therapeutics, College of Medicine, Jouf University, Sakaka 72388, Saudi Arabia; 3 Pharmacology Department, Faculty of Medicine, Mansoura University, Mansoura 35516, Egypt

**Keywords:** acute lymphoblastic leukemia, CAR T-cells, cytokine release syndrome, refractoriness/relapse

## Abstract

The currently available treatment for acute lymphoblastic leukemia (ALL) is mainly dependent on the combination of chemotherapy, steroids, and allogeneic stem cell transplantation. However, refractoriness and relapse (R/R) after initial complete remission may reach up to 20% in pediatrics. This percentage may even reach 60% in adults. To overcome R/R, a new therapeutic approach was developed using what is called chimeric antigen receptor-modified (CAR) T-cell therapy. The Food and Drug Administration (FDA) in the United States has so far approved four CAR T-cells for the treatment of ALL. Using this new therapeutic strategy has shown a remarkable success in treating R/R ALL. However, the use of CAR T-cells is expensive, has many imitations, and is associated with some adverse effects. Cytokine release syndrome (CRS) and immune effector cell-associated neurotoxicity syndrome (ICANS) are two common examples of these adverse effects. Moreover, R/R to CAR T-cell therapy can take place during treatment. Continuous development of this therapeutic strategy is ongoing to overcome these limitations and adverse effects. The present article overviews the use of CAR T-cell in the treatment of ALL, summarizing the results of relevant clinical trials and discussing future prospects intended to improve the efficacy of this therapeutic strategy and overcome its limitations.

## 1. Introduction

Acute lymphoblastic leukemia (ALL) is a rare cancer in which there is an abnormal clonal proliferation of early lymphoid stem cells along with the depletion of normal bone marrow hematopoietic cells [1]. The two major types of ALL are B and T lymphoblastic leukemia. While precursor B-cell ALL (B-ALL) represents the majority of ALL cases, T-cell neoplasm tends to behave more aggressively [2]. In 2021, around 6000 cases of ALL were reported in the United States. Children and young adults’ affection represent around 80% of these cases [3]. Combination of chemotherapy, steroids, and allogeneic stem cell transplantation (Allo-SCT) is the mainstay for treating this type of leukemia [4]. While such therapeutic protocols were able to increase long-term survival rates to around 90% in pediatric ALL [5], refractoriness and relapse (R/R) after initial complete remission (CR) were reported in up to 20% of the cases [2,6]. In comparison to pediatric ALL, the figures are worse in adult leukemia. It was reported that the percentage of adults with ALL who achieved long-term disease-free survival was only 40% [7]. After relapse, the reported overall survival was only about 7% [8]. Such facts urge the need for newer therapeutic approaches to overcome R/R. One of these approaches that has been developed over the past few years is the use of chimeric antigen receptor-modified (CAR) T-cell therapy.

## 2. What Is CAR T-Cell Therapy?

CAR T-cells were developed and proposed as a potential therapeutic strategy by Gross and colleagues more than 3 decades ago [9]. The use of these cells entails the genetic modification of T-cells in order to enhance their ability to express cell surface synthetic receptors which are capable of interacting with specific antigens expressed on the cancer cells [10]. CARs are fusion proteins. The primary structure of a CAR consists of an extracellular tumor antigen-binding domain called the ectodomain, a transmembrane domain, and an endodomain which is an intracellular signaling domain responsible for T-cell activation [10,11]. The striking difference between CAR T-cells and unmodified T-cells is that CAR T-cells do not require human major histocompatibility complex (MHC) molecules to recognize antigens on the surface of tumor cells. CAR T-cells have the ability to directly attack cancer once these cells bind to the specified antigen located on the surface of tumor cells [10].

Currently, there are four generations of CAR T-cells. An additional fifth generation is in the pipeline. The design of the first generation of CAR T-cells was simple. The endodomain responsible for the activation of T-cells was formed of CD3ζ chain only [12]. Such a simple structure enabled only the activation of T-cells without being capable of supporting their cellular expansion. Thus, the therapeutic efficacy of this early generation was limited [9]. In fact, three signals are required to optimize T-cell activation and proliferation. Using CD3ζ chains in the first generation only ensure signal 1, which is known as T-cell receptor engagement. The other two signals are co-stimulation, which is called signal 2, and cytokine engagement, which is known as signal 3 [13]. The lack of co-stimulation (signal 2) was then addressed in the second and third generations of CAR T-cells. The addition of one or two co-stimulatory signaling domains to the endodomain of the second and third generation, respectively, fulfilled the function of signal 2 [9,14]. The use of the signaling domains of CD28, CD134, or CD137 incorporated to CD3ζ chains has significantly enhanced the activity, expansion, and survival of these modified T-cells and even stimulated interleukin (IL)-2 production [15,16,17]. The fourth generation is much more sophisticated. Modification of the structure to allow remote-controlling of CAR T-cells activity and limiting their toxicity is one example [18]. Modifications to overcome target antigen loss or down-regulation, which results in tumor escape [19,20], or to increase the CAR T-cell specificity by requiring the presence of two antigens on the same cell to allow T-cell activation [21,22], have also been reported. CAR T-cells may also be modified to enhance their ability to produce inducible cytokines, as IL-12 or interferon (IFN)-γ, which can modulate the tumor microenvironment and enhance CAR T-cells function [23,24]. These cells are known as CAR T-cells redirected for universal cytokine killings (TRUCKs), and they have demonstrated a significant ability to overcome target antigen loss/antigen-negative relapse on the surface of cancer cells [23]. The fifth generation is still in the exploratory stage. This generation is designed to cover cytokine engagement (signal 3). CAR T-cells belonging to this generation have the ability of inducing cytokine signaling upon antigen stimulation. CAR T-cells of this generation encode a truncated cytoplasmic domain from the IL-2 receptor β-chain with a transcription factor. These cells showed antigen-dependent activation and superior antitumor effects with significantly minimized toxicities. It is also expected that these cells would have a better outcome against antigen-positive relapse [25,26]. Of note, none of the third, fourth, or fifth generations of these modified T-cells have been approved yet for clinical use.

## 3. Manufacturing of CAR T-Cells

The manufacturing of CAR T-cells starts first with the isolation of peripheral blood mononuclear cells from the patient (autologous) or a healthy donor (allogeneic) using leukapheresis. The next step entails the use of specific antibodies to separate the target T-cell subsets, which are then activated by beads coated with anti-CD3/anti-CD28 monoclonal antibodies, anti-CD3 antibodies, or artificial antigen-presenting cells (APCs). The activation process may be enhanced by cytokines, such as IL-2. The following step is the process of gene transfer of the CAR construct to the primary T-cells. Owing to the lower risk of mutagenesis, lentiviral vectors are preferred for gene transfer over retroviral vectors [27,28]. Non-viral approaches for gene transfer have also been used in this step. The designated CAR T-cells are subjected to expansion and phenotyping, and then they will be cryopreserved for subsequent infusion [28,29,30].

## 4. CAR T-Cells for ALL

There are several antigens on ALL cells that have been targeted in different experimental and clinical trials on CAR T-cell therapy. CD19 has received most of the attention in B-ALL. It is constantly expressed throughout B-cell development. In addition, CD19 is not expressed on hematopoietic stem cells. This will reduce the chance of developing aplastic anemia while using this therapeutic strategy [31]. Still, targeting CD19 could possibly result in B-cell aplasia with a consequent long-lasting defect in humoral immunity and recurrent respiratory tract infections [32,33]. At the moment, all the currently FDA-approved CAR T-cells for treating ALL are targeting CD19 (Table 1). 

CD20 is another target antigen under investigation as it is specific for B-cell lymphoid malignancies [38,39]. Similarly, the receptor for thymic stromal lymphopoietin (TSLPR) was also considered as a promising target for CAR T-cell therapy in selected cases of B-ALL [40,41]. For T-cell ALL, CD7-based CAR T-cells are being investigated in xenograft models, and promising results have encouraged an ongoing clinical trial [42,43]. Similarly, CD38-based CAR T-cells have been designed to target HTLV-1 + T-cell leukemia [44,45,46]. Other than targeting only one antigen on the surface of ALL cells, the use of multiple targets, as in the fourth generation of CAR T-cells, is still under investigation in order to reduce on-target, off-tumor toxicities [21,22] and prevent tumor antigen-loss relapses [19,20].

## 5. Limitations and Toxicities of CAR T-Cell Therapy

The first barrier to accessing CAR T-cell therapy is probably the cost. In the United States, it was estimated that one infusion of CAR T-cells can cost between USD 373,000 and 475,000 [47]. Patient care and disease monitoring can increase the number by USD 79,466 to 85,267 [48]. Moreover, the management of CAR T-cell toxicities and post-CAR T-cell therapy events can increase the total cost to exceed USD 1 million for some patients. As an average, the cost for this new therapeutic strategy can range from USD 500,000 to 600,000 [49,50]. Several technical and regulatory issues along with institutional infrastructure readiness might also be limiting factors for the use of CAR T-cell therapy [51]. Away from the high cost barrier, the two major limitations for CAR T-cell therapy are failure of treatment and toxicity.

### 5.1. Failure of CAR T-Cell Therapy

Treatment failure is a major limiting factor for the use of CAR T-cell therapy. The incidence of relapse among B-ALL patients who received treatment with CAR T-cells showed a wide variation in different studies and ranged between 22% and 75% [52]. Considering that only anti-CD19 CAR T-cells are the currently FDA-approved CAR T-cell therapy for B-ALL, we can classify relapse to either CD19-positive or CD19-negative relapses. Antigen-positive relapse is more common and is mainly related to T-cell potency and its in vivo expansion ability. Finding that the rate of B-ALL relapse is less in children compared to adult patients suggested that the age of T-cell might be a major determinant for antigen-positive relapse [53]. The use of co-stimulation/signaling domains and other techniques to enhance the activity, stimulate the expansion, and extend the survival of CAR T-cells has been suggested, and some have shown a promising ability to reduce antigen-positive relapse [17,26,54,55,56]. On the other hand, antigen-negative relapse or tumor antigen escape is more relevant to tumor cells. Generally, loss of CD19 antigen is responsible for about 10–20% of relapses in patients with B-ALL [57]. However, some clinical trials reported higher percentages [58]. Antigen-negative subclones might pre-exist even before starting CAR T-cell therapy and cause R/R [59]. Several mechanisms have been proposed to explain the antigen-negative relapse. While gene mutation, pre-treatment with blinatumomab, and RNA splice variants [60,61,62] can explain most of the cases of CD19-negative relapse, down-regulation of the target antigen can take place with other CAR T-cell therapies of B-ALL as anti-CD22 CAR T-cell therapy [63]. That is why the design of many of the fourth generation CAR T-cells is addressing the relapse due to antigen loss. Designing CAR T-cells with dual CD19/CD123 targeting was reported to prevent antigen-negative relapses [20]. Still, the genetic instability and heterogeneity of antigen expression on the leukemic cell surface, especially in the case of high tumor burden, can lead to another relapse even after dual-target CAR T-cell therapy [64,65,66]. Other B-cell lineage markers, as CD20 and CD22, have also been targeted, and epigenetic modifying agents have also been tested to overcome the immune evasion of tumor cells during therapy with CAR T-cells [67,68,69].

### 5.2. Cytokine Release Syndrome

The toxicity of CAR T-cell therapy can take many forms. One of the most important and most common toxicities related to the use of this relatively new therapeutic strategy is the cytokine release syndrome (CRS) [70]. In children with B-ALL, it was reported that the incidence of CRS can reach up to 77% [58]. In adults, figures are much higher and ranged from 85 to 93% depending on the study [53,71]. In CRS, the activation of T-cells will lead to a significant elevation of the inflammatory cytokines (e.g., IL-6, IL-10, IFN-γ, and GM-CSF) [72,73,74]. Patients are usually presented with fever, headache, myalgia, hypotension, and hypoxia. The condition may be complicated with cardio-respiratory dysfunction or even failure. The burden of the disease and the used dose of CAR T-cells are major determinant of the severity of the CRS [72,73]. The management of CRS is largely dependent on the disease grading. Anti-cytokine therapy is the mainstay for treating CRS together with proper supportive treatment for the associated hypotension and/or hypoxia. IL-6 receptor blockade via tocilizumab with or without systemic corticosteroids is usually needed in grade 2 CRS and onward. Siltuximab, an anti-IL-6 monoclonal antibody, and anakinra, an IL-1 receptor antagonist, have been suggested as alternatives to tocilizumab. Most patients respond well after therapy, yet CRS symptoms may persist in very rare cases [75,76,77].

### 5.3. Immune Effector Cell-Associated Neurotoxicity Syndrome

Another common toxicity associated with the use of CAR T-cells is known as immune effector cell-associated neurotoxicity syndrome (ICANS). Patients may show a wide range of presentation varying from confusion and delirium to motor dysfunction, expressive aphasia, tremor, and ataxia. Rare cases presented with myoclonic seizure and even fatal brain edema [72,73,74]. The incidences of these presentations also vary widely between studies and are largely affected by the used grading scale [78,79]. The precise mechanism of these neurologic adverse effects is unknown, but it is likely to be related to the elevated levels of different inflammatory cytokines. That is why such syndrome usually follows CRS [80]. As CD19 is expressed on the left inferior frontal gyrus, it is possible that ICANS is part of the on-target/off-tumor effect [81]. However, reporting similar neurotoxicity with CAR T-cells targeting other antigens may suggest that ICANS is not antigen-specific [82,83]. The disease is self-limited and may resolve even without any intervention [84]. Unlike CRS, tocilizumab has no role in ICANS and may even worsen the neurological manifestations. If needed, treatment with corticosteroids in addition to standard care treatment for neurological presentation, such as seizures, can reverse the disease in the majority of patients. Still, few mortalities have been reported with ICANS, especially when the cases were associated with cerebral edema [75,76,77]. Of note, both CRS and ICANS are more likely to take place when anti-CD19 CAR T-cells were used for B-ALL in comparison to their usage in other tumors [85].

### 5.4. Infection and Hypogammaglobulinemia

Infection is another complication to the use of CAR T-cells in leukemia. The reported incidence of infection with CAR T-cell therapy is around 43% [58]. Early infection is usually bacterial and is probably due to the immunosuppressive effect of lymphodepleting chemotherapy which is given before CAR T-cell infusion. Late infection is more relevant to CAR T-cell therapy and is more likely to be viral. This is attributed to CAR T-cell therapy itself in a phenomenon called “on-target/off-tumor recognition”. As the target antigen for CAR T-cell is shared by normal cells, it is possible that non-pathogenic immune cells expressing the target antigen (CD19) will also be affected by the used CAR T-cells. The delayed recovery of these cells will result in significant hypogammaglobulinemia [86,87,88]. The condition could be fatal and requires IgG replacement to protect against infectious complications [86,88].

### 5.5. Other Toxicities of CAR T-Cell Therapy

Another immunological dysfunction that might take place after CAR T-cell therapy is called hemophagocytic histiocytosis (carHLH) [75,89]. The mechanism of carHLH is unknown but involves the production of pro-inflammatory cytokines, lymphohistiocytic tissue infiltration, and immune-induced multiorgan damage and dysfunction. Until the proper patient identification and management protocols are standardized, the current management of carHLH is identical to severe CRS cases [77]. Other toxicities that have been reported with CAR T-cell therapy include cerebral edema, cardiotoxicity, allergic reaction, and tumor lysis syndrome [77].

## 6. Clinical Trials Using CAR T-Cells in ALL

A long list of clinical trials which are investigating the efficacy and safety of CAR T-cells in the treatment of ALL are ongoing/recruiting, completed, or even withdrawn [85,90]. We have listed the results of some of these clinical trials in Table 2. Other than clinical trials, several studies have been published to evaluate collective data obtained from related previous clinical trials. One example is the study of Pasquini et al. [91], who collected the safety and efficacy data of a CD19-targeting CAR T-cell therapy: tisagenlecleucel (tisa-cel/Kymriah). The data of 410 patients have been evaluated. There were 255 ALL patients, while the remaining 155 were non-Hodgkin lymphoma patients. The initial cure rate among ALL patients was 85.5%. Overall survival at 12 months was 77.2%. Adverse effects as grade-3 CRS accounted for 16.1% of the patients, while grade-3 ICANS was about 9.0%. These figures are much lower than those reported in some of the key studies, probably due to using different grading scales for the assessment of these adverse effects. In a parallel context, we have found one meta-analysis of several clinical trials that reported the safety and efficacy data of anti-CD19 CAR T-cells. Pooled data of 953 R/R B-ALL patients were presented, and the pooled CR was about 80%. Of note, CR was significantly improved when autologous CAR T-cells were used in comparison to allogeneic CAR T-cells. However, using autologous CAR T-cells was more likely to cause ICANS. In addition, the percentages of patients having CRS or ICANS were not significantly affected by the use of different anti-CD19 CAR T-cell constructs [92].

Of note, most of the clinical trials listed in Table 2 were using CD19-targeting CAR T-cells; few trials tested other targets. Investigating other targets would be helpful in case of relapse after using CD19-targeting CAR T-cells. In the study of Pan and his colleagues, they used CD22-targeting CAR T-cells in patients with B-ALL who failed to respond to CD19 CAR T-cell therapy. Among the 34 enrolled patients, 24 patients achieved CR or CR with incomplete count recovery after 30 days. Seven CR patients did not receive any additional treatment, and three of these patients remained in remission after 14 months. Transplantation was conducted in 11 CR patients, and 8 of them remained in remission at 4.6 to 13.3 months. Adverse effects were mild in most of the patients, and CD22 antigen mutation or loss was not reported even in patients with reported relapse [93]. Using dual-target CAR T-cells was also subjected to clinical testing to overcome relapses that might take place after the use of CD19-targeting CAR T-cell therapy. In one study, bispecific CAR T-cells targeting both CD19 and CD22 were used in patients with R/R B-ALL. All the six enrolled patients achieved CR. No neurotoxicity was reported, but one case of relapse took place 5 months later where the blast cells of the patients lost CD19 expression and exhibited diminished CD22 site density [94]. Subsequent studies enrolled more patients to enhance the reliability of the results [95,96]. A recently published meta-analysis evaluated the efficacy and safety of CD22 or CD19/CD22 CAR T-cells in the treatment of ALL and NHL. In ALL, CD19/CD22 CAR T-cells had a CR rate of about 90%. The incidences of total and severe CRS were 87% and 6%, respectively. The incidences of ICANS and severe ICANS were 16% and 3%, respectively [97].

**Table 2:** Selected clinical trials that investigated the safety and efficacy of using CAR T-cells in the treatment of acute lymphoblastic leukemia (ALL).

Clinical Trial	Age, Number, Type of Leukemia	Follow Up (Months)	Used CAR T-Cell	T-Cell Persistence	CR, OS	RELAPSE RATE	Reported Adverse Effects	Reference
NCT01626495 and NCT01029366	1–24 years, 30, R/R ALL	6	CD19-targeting autologous CAR T-cells (CTL019)	68% at 6 months	90%, 78%	26%	100% CRS and 27% severe CRS 43% total neurologic toxicity	[73]
NCT01626495	5–22 years, 39, R/R ALL		CD19-targeting autologous CAR T-cells (CTL019)				46% CRS, hyperferritinemia, and organ dysfunction. 36% cardiovascular dysfunction 15% acute respiratory failure	[98]
NCT01593696	1–30 years, 21, R/R B-ALL or NHL	10	CD19-CAR incorporating an anti-CD19 single-chain variable fragment + CD3ζ + CD28 signaling domains	0% at 6 months	67%, 51.6 at 10 months	17%	76% CRS 14% sever CRS 43% fever 43% hypokalemia 38% fever and neutropenia	[74]
NCT01593696	4.3–30.4 years, 50, R/R B-ALL	57	CD19-CAR incorporating an anti-CD19 single-chain variable fragment + CD3ζ + CD28 signaling domains		62%, Nr	9.5% at 24 months		[99]
NCT02030847 NCT01029366	20.6–70.4, 35, R/R B-ALL	13	anti-CD19 CAR T-cells therapy tisagenlecleucel (CTL019)		90%, 73% in the high dose group		90% total CRS 40% total neurologic toxicity	[100]
NCT03289455 Amelia study	1–24, 15, R/R B-ALL	14	Simultaneous targeting of CD19 and CD22 (AUTO3)		86%, 60% at one year		80% mild CRS 7% encephalopathy	[95]
NCT03289455	27–83, 52, R/R large B-ALL	21.6	Simultaneous targeting of CD19 and CD22 (AUTO3) plus pembrolizumab		48.9%		34.6% grade 1–2 CRS 1.9% grade 3 CRS 8% neurotoxicity	[96]
NCT02975687	3–52 years, 20, R/R ALL	10.09	CD 19	6 months detected	90%		95% CRS	[101]
NCT02435849 ELIANA study	3–30, 75 + 4, R/R ALL	13	CTL019 Tisagenleucel (Tisacel) An anti-CD19 single-chain variable fragment + CD3ζ + co-stimulatory signals (4-1BB).	83% at 6 months	81%, 76% at 12 months	33%	77% CRS 44% sever CRS 40% ICANS 13% severe ICANS	[58,102]
Follow up of ELIANA study	0–26, 185, R/R ALL	11.2	Tisacel		75% and 63% at 6 and 12 months Os 85% at 6 months and 72% at 12 months	35%	60% CRS of any grade 22% neurotoxicity of any grade 19% ≥ grade 3 CRS 7% ≥ grade 3 neurotoxicity One grade 5 CRS and 1 grade 5 neurotoxicity (intracranial hemorrhage).	[103]
NCT01044069 MSKCC study	23–74, 53, R/R ALL	29	CD19 autologous T-cells expressing the 19-28z CAR	0% at 6 months/	83%, 12.9 months (median)	39%	26% severe CRS One patient died	[53,72]
NCT02028455	1–26, 45, ALL	9.6	SCRI-CAR19v1 (a CD19 specific CAR T-cell product)	~30% at 6 Months	89%, 70% at 12 months	45%	90% CRS 23% severe CRS 49% ICANS 23% severe ICANS	[104]
NCT01860937	1–22.5, 25, R/R B-ALL		CD19 19-28z CAR T-cells		75%, Nr		80% CRS 16% severe CRS 28% sever neurotoxicity	[105]
NCT01865617	20–47 years, 53, R/R B-ALL	30.9	Autologous CD19 CAR T-cells with a 4-1BB co-stimulatory		93% 85% long term, Nr	33%	23% CRS	[106,107]
NCT02443831	0–24, 14, different hematological malignancies		CD19	78.5% at 7 months	86%, 70%	40–60%	93% CRS	[108,109]
NCT03825731	1–45, 17 (4 adults, 13 pediatrics), R/R B-ALL		Anti-CD19/CD22 dual CAR-T		100% CR initially, Nr	50% of patients relapse at 1 year	94% had grade 0–1 CRS 5.88% grade 2 CRS	[110]
NCT03330691	Up to 30 years, 27, R/R ALL		CD19- or CD22-specific CAR T-cell construct. Both with 4-1BB co-stimulation.		84.6%	4 cases (15.4%)	80% CRS (85% grade 2 or less) 38% mild neurotoxicity with a single grade 3 event.	[111]
NCT03173417	2–14 years (61 patients) and 15–61 years (39 patients) R/R B-ALL, including high-risk features: *BCR-ABL* fusion gene, TP53 mutation (12), extramedullary disease (including patients with central nervous system leukemia), and relapses after allogeneic stem cell transplantation (16)	8	Anti-CD19 with 4-1BB and CD3ζ cytoplasmic domains		93% OS at 12 months was 64% OS were lower for patients carrying the TP53 mutation or history of previous transplantation		Grade 3 and 4 CRS were 16.9% and 15.4% Grade 2 to 3 neurological events were 12.7% and 15.4%	[112]
NCT02614066 Zuma 3	28–52, 55, R/R B-ALL	16.4	Autologous anti-CD19 CAR T-cell, KTE-X19 (Brexucabtagene Autoleucel): anti-CD19 single-chain variable fragment + CD3ζ + CD28 signaling domains		71% had CR/Cri and 51% CR, OS 12.8 months		Of grade 3 or higher: 49% anemia 36% pyrexia 25% infections 24% CRS 25% neurological events Two grade 5 KTE-X19-related events (brain herniation and septic shock).	[113]
NCT02625480 Zuma 4	3–20, 24, R/R B-ALL	36.1	KTE-X19 (Brexucabtagene Autoleucel)		67% had CR/Cri, Nr		Grade ≥3 adverse events in all patients: 50% hypotension 42% anemia 33% grade 3 CRS 21%, 25%, 27%, and 11% neurological events depending on the dose	[114]

ALL: acute lymphoblastic leukemia; CR: complete remission; Cri: complete remission with incomplete count recovery; CRS: cytokine release syndrome; ICANS: immune effector cell-associated neurotoxicity syndrome; NHL: non-Hodgkin lymphoma; Nr: not reported; OS: overall survival; R/R: relapsed/refractory. N.B. Whenever more than one article described the same clinical trial, we used to include the results of the latest one and point to relevant articles where preliminary data were described.

## 7. Future Prospects for CAR T-Cell Therapy in the Treatment of ALL

Although three decades have now lapsed since CAR T-cell therapy was first introduced as a new therapeutic modality, efforts to improve this therapeutic approach are still ongoing to overcome its limitations. One of these limitations is the time needed to manufacture personalized T-cells. Such a process can take up to 3 weeks and delay treatment [115,116]. This has led to the development of what is known as “off-the-shelf” CAR T-cells.

Off-the-shelf CAR T-cells are newly developed universal allogeneic CAR T-cells where T-cells are collected from healthy donors. This ensures that a good number of healthy T-cells are retrieved and, unlike autologous CAR T-cells, are not affected by chemotherapy or cancer cells [117,118]. It was suggested that CAR T-cells performance could be greatly affected by the quality and quantity of retrieved T-cells [53,119]. Thus, we might expect that the performance of such produced allogeneic CAR T-cells would be improved in comparison to autologous cells. Off-the-shelf CAR T-cells, in addition to ensuring good-quality cells, will also facilitate the large-scale production of T-cells, which saves both money and time. It allows the patient to have immediate access to the treatment. There is no need to wait for the 3-week interval which is usually required to manufacture personalized autologous CAR T-cells [115,118]. The term (FasTCAR) is gaining popularity and is used to indicate the next-day manufacturing of CAR T-cells using novel manufacturing platforms. The results of clinical trials on patients with relapsed ALL indicated the superior expansion capacity of these allogeneic CAR T-cells and comparable efficiency with manageable toxicity profile [120]. However, one meta-analysis reported that while neurotoxicity was reduced with allogeneic CAR T-cells, CR rates were higher when autologous CAR T-cells were used in comparison to allogeneic CAR T-cells [92]. In addition, there is another major drawback of off-the-shelf CAR T-cells. Using T-cells from a donor with MHC-mismatching can be complicated with graft rejection and the serious and life-threatening complication, graft-versus-host disease (GVHD).

In graft rejection, the remaining functioning host immune cells will attack and eliminate the transferred CAR T-cells and compromise their antitumor efficacy [121]. Rejection of allogenic CAR T-cells can be prevented in several ways. One way is to disrupt certain components of the MHC Class I of the allogeneic CAR T-cell. This might prevent the allogenic rejection by the host cells [122,123,124]. Another approach is to delete CD52 on the surface of CAR T-cells. At the same time, the anti-CD52 monoclonal antibody, alemtuzumab, is used to deplete the host T-cells before CAR T-cell infusion. Thus, the infused T-cells evaded the rejection and simultaneously will not be targeted by alemtuzumab. Such a strategy was successful but was hindered by the increased risk of cytopenia and severe viral infections [125,126]. Another way to prevent the rejection of transferred allogenic CAR T-cells is to engineer a chimeric receptor, called the alloimmune defense receptor (ADR), that enables used CAR T-cells to recognize and eliminate alloreactive lymphocytes while saving the host resting lymphocytes in peripheral blood. It was found that the co-expression of ADR and CAR on the engineered T-cells conveyed resistance to allogenic rejection, while the anti-tumor activity was not compromised [121,127].

On the other side, different strategies have been tested to bypass GVHD. Using third-party allogeneic virus-specific T-cells is one example that has been suggested based on the encouraging data when these cells were used to treat post-transplant viral infections [128,129,130,131]. Another method to overcome GVHD is the use of T-cells that are genetically modified. The genetic modification of T-cells entails the removal of endogenous molecules, as αβ T-cell receptors, in order to make the produced T-cells unable to mount the usual alloreactive immune response against the recipient’s normal tissue. Genetic modification could be achieved using different gene editing techniques, such as clustered regularly interspaced short palindromic repeat (CRISPR)-associated proteins or transcription activator-like effector nucleases (TALENS) [122,123,124,132]. Such a strategy showed some success in small clinical studies [125,126,133]. Instead of removing the αβ T-cell receptors, using a minor T-cell subset called γδ T-cells could be also promising to overcome GVHD. These cells represent only 5–10% of the total population of T-cells but have a striking feature in the way that the expression of γδ T-cell receptors on the surface of these cells is MHC-independent. This feature made this subtype of T-cells a suitable candidate for off-the-shelf CAR T-cell production with minimal risk of GVHD [134].

Another strategy to circumvent GVHD, other limitations, and the toxicities of CAR T-cells is to replace T-cells with an alternative effector cell. In this context, Natural killer (NK) cells and macrophages might be perfect targets for producing off-the-shelf CARs. These cells, unlike T-cells, are components of innate immunity, and their action is independent of MHC. Thus, it is unlikely that they may cause GVHD. Moreover, antigen escape is also unlikely to take place with NK-based CARs. These cells have the ability to recognize cancer cells independent of MHC and will keep their potency even if MHC molecules are down-regulated [135]. Indeed, CAR-NKs have shown promising results with minimal toxicities in several models of hematological and solid tumors, as well as one clinical trial [136]. Several other clinical studies have been registered, though the results are yet to be announced [137]. For macrophages, they have several advantages that can make them suitable for CARs. These cells, by selective phagocytosis, destroy cancer cells and then activate adaptive immunity by presenting the antigens to T-cells. Macrophages, by enhancing the secretion of pro-inflammatory cytokines, can also potentiate the cytotoxicity of T-cells against tumors [138,139]. In addition, these cells can easily infiltrate the tumor microenvironment and secrete cytokines that modulate the suppressive microenvironment generated by tumor cells [139]. Macrophages-based CARs showed some success in preclinical studies, and clinical trials are being conducted [138,140].

Future prospects in CAR T-cell therapy also entail the search for newer targets. As we previously discussed, all the currently available treatments for B-ALL are based on targeting CD19. Thus, tumor relapse is a common limitation for all currently available CAR T-cell therapy as a result of the down-regulation of CD19 on the surface of cancer cells [57,58]. For this reason, many of the recent studies actually focused on the search for new targets, such as CD20, CD22, and BCMA [67,69,82,141]. The success of these newer targets in comparison to targeting CD19 remains questionable. It was suggested that CD19 expression is more stable than other targets. Such stability could allow for obtaining better antitumor activity in comparison to other antigens [142]. However, one clinical trial was conducted on B-ALL patients using a relatively low dosage of CD20 CAR T-cell therapy. The study reported that targeting CD20 resulted in the induction of a high remission rate and low toxicities in patients who relapsed after CD19 CAR T-cell therapy [66]. Another new method to avoid CD19-negative relapses is to use dual-target CARs. The dual-target CARs strategy entails simultaneous targeting CD19 and another antigen. For example, the presence of either CD19 “OR” CD20 on target cells can fully induce the immune response of anti-CD20-CD19 bispecific CAR T-cells in preclinical settings [143]. Bispecific CAR T-cells could also be designed to work upon the simultaneous presence of both antigens, for example, CD19 “AND” CD22 [94,144,145]. The dual targeting of CD19 and CD123 or B-cell activating factor receptor (BAFF-R) has also been tested [146,147]. CAR T-cells which are targeting three antigens, CD19/CD20/CD22, are an even more sophisticated strategy being tested for the treatment of ALL. Initial results showed that these trivalent CAR T-cells were more effective in killing ALL cells, including CD19-negative ones [148]. In a parallel context and rather than using dual/trivalent targeting CAR T-cells, Yan et al. used a sequential infusion of two different CAR T-cells, anti-CD19 and anti-CD22, in patients with relapsed ALL after hematopoietic stem cell transplantation (HSCT). This trial has shown that the use of such a cocktail was safe and effective for relapsed cases of ALL [149].

## 8. CAR T-Cell Therapy for T-Cell Acute Lymphoblastic Leukemia (T-ALL)

Another indication for the use of CAR T-cell therapy is to target T-ALL. As we mentioned earlier, T-ALL is a rare but aggressive tumor with a very poor prognosis [2,150]. One major limitation that hindered the progress of developing CAR T-cells against T-ALL is that the T effector cells, which is used as a treatment, share the expression of almost all the antigens as malignant T-cells [151,152]. This will result in self-killing or what is known as the fratricide of CAR T-cells. Fratricide can reduce the proliferation of CAR T-cells in vitro and compromise their action after being infused. Several strategies have been tested to overcome this obstacle. As we discussed earlier, replacing T-cells with NK cells is one strategy. Several preclinical studies used CAR-modified NK cells to treat T-ALL, and the results were indeed encouraging to conduct clinical trials using these cells [153,154,155]. Another strategy to avoid fratricide is to find a tumor-specific antigen with a restricted expression to malignant T-cells only. As reviewed by Ren and his colleagues [156], several biomarkers have been identified and can be targeted by CAR T-cells depending on the limited expression of these biomarkers on normal T-cells. CD7 or CD38-based CAR T-cells are relevant examples [42,43,44,45,46]. While some studies showed a successful generation of anti-CD147-CAR T-cells that conveyed protection against T-ALL progression in a xenograft model [157], others have been tested clinically. For example, nanobody-derived CD7-CAR T-cells showed a remarkable ability to induce remission in one case of an 11-year-old male patient with R/R early T-ALL [158].

## 9. Conclusions

CAR T-cell therapy appears to be a promising new therapy for ALL, especially cases with R/R, as evident in several experimental and clinical settings. However, as with any therapeutic modality, using CAR T-cells is associated with several limitations and toxicities. In addition, the process of manufacturing these personalized T-cells is expensive and time-consuming. Continuous development of this therapeutic strategy is ongoing to overcome these limitations and adverse effects, reduce cost, and ensure the availability of this therapeutic approach in the proper time.

## Figures and Tables

**Table 1:** Currently FDA-approved CAR T-cell therapy for ALL [34,35,36,37].

Trade Name	Generic Name	FDA Approval Date	Target Antigen	Indication(s)
BREYANZI^®^	Lisocabtagene maraleucel	2021	CD19	For adult patients with R/R large B-cell lymphoma after two or more lines of systemic therapy, including: ○ Diffuse large B-cell lymphoma (DLBCL) not otherwise specified (including DLBCL arising from indolent lymphoma); ○ High-grade B-cell lymphoma; ○ Primary mediastinal large B-cell lymphoma; ○ Follicular lymphoma (FL) grade 3B.
KYMRIAH^TM^	Tisagenlecleucel	2017	CD19	For adults with R/R DLBCL. For young adult patients up to age 25 with R/R ALL. Adult patients with R/R FL after two or more lines of systemic therapy.
TECARTUS^TM^	Brexucabtagene autoleucel	2020	CD19	For adult patients with R/R B-ALL. For patients with R/R mantle cell lymphoma.
YESCARTA^TM^	Axicabtagene ciloleucel	2017	CD19	For patients with the following conditions who have R/R following two or more lines of systemic therapy: ○ DLBCL; ○ Primary mediastinal B-cell lymphoma; ○ High grade B-cell lymphoma; ○ DLBCL that results from FL; ○ FL.
N.B. None of these therapeutics could be used to treat patients with primary central nervous system lymphoma. DLBCL: diffuse large B-cell lymphoma; FL: follicular lymphoma; R/R: relapsed/refractory.				

## Data Availability

Not applicable.

## References

[1] Malard F., Mohty M. (2020). Acute lymphoblastic leukaemia. Lancet.

[2] Hunger S.P., Mullighan C.G. (2015). Acute lymphoblastic leukemia in children. N. Engl. J. Med..

[3] Siegel R.L., Miller K.D., Fuchs H., Jemal A. (2021). Cancer statistics, 2021. CA Cancer. J. Clin..

[4] Terwilliger T., Abdul-Hay M. (2017). Acute lymphoblastic leukemia: A comprehensive review and 2017 update. Blood Cancer J..

[5] Pui C.H., Yang J.J., Hunger S.P., Pieters R., Schrappe M., Biondi A., Vora A., Baruchel A., Silverman L.B., Schmiegelow K. (2015). Childhood acute lymphoblastic leukemia: Progress through collaboration. J. Clin. Oncol..

[6] Hunger S.P., Raetz E.A. (2020). How I treat relapsed acute lymphoblastic leukemia in the pediatric population. Blood.

[7] Kantarjian H., Thomas D., O’Brien S., Cortes J., Giles F., Jeha S., Bueso-Ramos C.E., Pierce S., Shan J., Koller C. (2004). Longterm follow-up results of hyperfractionated cyclophosphamide, vincristine, doxorubicin, and dexamethasone (Hyper-CVAD), a dose-intensive regimen, in adult acute lymphocytic leukemia. Cancer.

[8] Fielding A.K., Richards S.M., Chopra R., Lazarus H.M., Litzow M.R., Buck G., Durrant I.J., Luger S.M., Marks D.I., Franklin I.M. (2007). The outcome of 609 adults after relapse of acute lymphoblastic leukemia (ALL); an MRC UKALL12/ECOG 2993 study. Blood.

[9] Gross G., Waks T., Eshhar Z. (1989). Expression of immunoglobulin-T-cell receptor chimeric molecules as functional receptors with antibody-type specificity. Proc. Natl. Acad. Sci. USA.

[10] Sadelain M., Brentjens R., Rivière I. (2013). The basic principles of chimeric antigen receptor design. Cancer Discov..

[11] Chen Y.-J., Abila B., Mostafa Kamel Y. (2023). CAR-T: What Is Next?. Cancers.

[12] Schubert M.L., Hoffmann J.-M., Dreger P., Müller-Tidow C., Schmitt M. (2018). Chimeric antigen receptor transduced T cells: Tuning up for the next generation. Int. J. Cancer..

[13] Kershaw M.H., Westwood J.A., Darcy P.K. (2013). Gene-engineered T cells for cancer therapy. Nat. Rev. Cancer.

[14] Wang J., Jensen M., Lin Y., Sui X., Chen E., Lindgren C.G., Till B., Raubitschek A., Forman S.J., Qian X. (2007). Optimizing adoptive polyclonal T cell immunotherapy of lymphomas, using a chimeric T cell receptor possessing CD28 and CD137 costimulatory domains. Hum. Gene Ther..

[15] Finney H.M., Lawson A.D., Bebbington C.R. (1998). Chimeric receptors providing both primary and costimulatory signaling in T cells from a single gene product. J. Immunol..

[16] Finney H.M., Akbar A.N. (2004). Activation of resting human primary T cells with chimeric receptors: Costimulation from CD28, inducible costimulator, CD134, and CD137 in series with signals from the TCRζ chain. J. Immunol..

[17] Carpenito C., Milone M.C., Hassan R., Simonet J.C., Lakhal M., Suhoski M.M., Varela-Rohena A., Haines K.M., Heitjan D.F., Albelda S.M. (2009). Control of large, established tumor xenografts with genetically retargeted human T cells containing CD28 and CD137 domains. Proc. Natl. Acad. Sci. USA.

[18] Wu C.Y., Roybal K.T., Puchner E.M., Onuffer J., Lim W.A. (2015). Remote control of therapeutic T cells through a small molecule-gated chimeric receptor. Science.

[19] Hegde M., Mukherjee M., Grada Z., Pignata A., Landi D., Navai S.A., Wakefield A., Fousek K., Bielamowicz K., Chow K.K. (2016). Tandem CAR T cells targeting HER2 and IL13Rα2 mitigate tumor antigen escape. J. Clin. Investig..

[20] Ruella M., Barrett D.M., Kenderian S.S., Shestova O., Hofmann T.J., Perazzelli J., Klichinsky M., Aikawa V., Nazimuddin F., Kozlowski M. (2016). Dual CD19 and CD123 targeting prevents antigen-loss relapses after CD19-directed immunotherapies. J. Clin. Investig..

[21] Lanitis E., Poussin M., Klattenhoff A.W., Song D., Sandaltzopoulos R., June C.H., Powell D.J. (2013). Chimeric antigen receptor T cells with dissociated signaling domains exhibit focused antitumor activity with reduced potential for toxicity in Vivo. Cancer Immunol. Res..

[22] Roybal K.T., Rupp L., Morsut L., Walker W.J., McNally K.A., Park J.S., Lim W.A. (2016). Precision tumor recognition by T cells with combinatorial Antigen-Sensing circuits. Cell.

[23] Li J., Li W., Huang K., Zhang Y., Kupfer G. (2018). Chimeric antigen receptor T cell (CAR-T) immunotherapy for solid tumors: Lessons learned and strategies for moving forward. J. Hematol. Oncol..

[24] Akhoundi M., Mohammadi M., Sahraei S.S., Sheykhhasan M., Fayazi N. (2021). CAR T cell therapy as a promising approach in cancer immunotherapy: Challenges and opportunities. Cell Oncol..

[25] Kagoya Y., Tanaka S., Guo T., Anczurowski M., Wang C.H., Saso K., Butler M.O., Minden M.D., Hirano N. (2018). A novel chimeric antigen receptor containing a JAK-STAT signaling domain mediates superior antitumor effects. Nat. Med..

[26] Zheng W., O’Hear C.E., Alli R., Basham J.H., Abdelsamed H.A., Palmer L.E., Jones L.L., Youngblood B., Geiger T.L. (2018). PI3K orchestration of the in vivo persistence of chimeric antigen receptor-modified T cells. Leukemia.

[27] Scholler J., Brady T.L., Binder-Scholl G., Hwang W.T., Plesa G., Hege K.M., Vogel A.N., Kalos M., Riley J.L., Deeks S.G. (2012). Decade-long safety and function of retroviral-modified chimeric antigen receptor T cells. Sci. Transl. Med..

[28] Levine B.L., Miskin J., Wonnacott K., Keir C. (2017). Global manufacturing of CAR T cell therapy. Mol. Ther. Methods. Clin. Dev..

[29] Johnson L.A., June C.H. (2017). Driving gene-engineered T cell immunotherapy of cancer. Cell Res..

[30] Sheykhhasan M., Manoochehri H., Dama P. (2022). Use of CAR T-cell for acute lymphoblastic leukemia (ALL) treatment: A review study. Cancer Gene Ther..

[31] Forsberg M.H., Das A., Saha K. (2018). The potential of CAR T therapy for relapsed or refractory pediatric and young adult B-cell ALL. Ther. Clin. Risk. Manag..

[32] Brudno J.N., Kochenderfer J.N. (2016). Toxicities of chimeric antigen receptor T cells: Recognition and management. Blood.

[33] Cordeiro A., Bezerra E.D., Hill J.A., Turtle C.J., Maloney D.G., Bar M. (2018). Late effects of CD19-targeted CAR-T cell therapy. Blood.

[34] FDA (2023). Package Insert-BREYANZI. https://www.fda.gov/media/145711/download.

[35] FDA (2023). Package Insert-KYMRIAH. https://www.fda.gov/media/107296/download.

[36] FDA (2023). Package Insert-TECARTUS. https://www.fda.gov/media/140409/download.

[37] FDA (2023). Package Insert-YESCARTA. https://www.fda.gov/media/108377/download.

[38] Zah E., Lin M.Y., Silva-Benedict A., Jensen M.C., Chen Y.Y.T. (2016). Cells expressing CD19/CD20 bispecific chimeric antigen receptors prevent antigen escape by malignant B cells. Cancer Immunol. Res..

[39] Riaz I.B., Zahid U., Kamal M.U., Husnain M., McBride A., Hua A., Hamadani A.A., George L., Zeeshan A., Sipra Q.R. (2017). Anti-CD 19 and anti-CD 20 CAR-modified T cells for B-cell malignancies: A systematic review and meta-analysis. Immunotherapy.

[40] Qin H., Cho M., Haso W., Zhang L., Tasian S.K., Oo H.Z., Negri G.L., Lin Y., Zou J., Mallon B.S. (2015). Eradication of B-ALL using chimeric antigen receptor—Expressing T cells targeting the TSLPR oncoprotein. Blood.

[41] Davies D.M., Maher J. (2015). TLSPR: A new CAR in the showroom for B-ALL. Blood.

[42] Gomes-Silva D., Srinivasan M., Sharma S., Lee C.M., Wagner D.L., Davis T.H., Rouce R.H., Bao G., Brenner M.K., Mamonkin M. (2017). CD7-edited T cells expressing a CD7-specific CAR for the therapy of T-cell malignancies. Blood.

[43] Fleischer L.C., Spencer H.T., Raikar S.S. (2019). Targeting T cell malignancies using CAR-based immunotherapy: Challenges and potential solutions. J. Hematol. Oncol..

[44] Mihara K., Yoshida T., Ishida S., Takei Y., Kitanaka A., Shimoda K., Morishita K., Takihara Y., Ichinohe T. (2016). All-trans retinoic acid and interferon-α increase CD38 expression on adult T-cell leukemia cells and sensitize them to T cells bearing anti-CD38 chimeric antigen receptors. Blood Cancer J..

[45] Smith A.J., Oertle J., Warren D., Prato D. (2016). Chimeric antigen receptor (CAR) T cell therapy for malignant cancers: Summary and perspective. J. Cell Immunother..

[46] Hofmann S., Schubert M.-L., Wang L., He B., Neuber B., Dreger P., Müller-Tidow C., Schmitt M. (2019). Chimeric antigen receptor (CAR) T cell therapy in acute myeloid leukemia (AML). J. Clin. Med..

[47] Fiorenza S., Ritchie D.S., Ramsey S.D., Turtle C.J., Roth J.A. (2020). Value and affordability of CAR T-cell therapy in the United States. Bone Marrow Transpl..

[48] Lyman G.H., Nguyen A., Snyder S., Gitlin M., Chung K.C. (2020). Economic evaluation of chimeric antigen receptor T-cell therapy by site of care among patients with relapsed or refractory large B-cell lymphoma. JAMA Netw. Open..

[49] Hernandez I., Prasad V., Gellad W.F. (2018). Total costs of chimeric antigen receptor T-cell immunotherapy. JAMA Oncol..

[50] Di M., Long J.B., Isufi I., Foss F.M., Seropian S., Gross C.P., Huntington S.F. (2022). Total costs of care during chimeric antigen receptor T-cell therapy in patients with relapsed/refractory B cell non-Hodgkin lymphoma: A large private insurance claim-based analysis. Blood.

[51] Gee A.P. (2015). Manufacturing genetically modified T cells for clinical trials. Cancer Gene Ther..

[52] Fabrizio V.A., Curran K.J. (2021). Clinical experience of CAR T cells for B cell acute lymphoblastic leukemia. Best Pract. Res. Clin. Haematol..

[53] Park J.H., Rivière I., Gonen M., Wang X., Sénéchal B., Curran K.J., Sauter C., Wang Y., Santomasso B., Mead E. (2018). Long-term follow-up of CD19 CAR therapy in acute lymphoblastic leukemia. N. Engl. J. Med..

[54] Long A.H., Haso W.M., Shern J.F., Wanhainen K.M., Murgai M., Ingaramo M., Smith J.P., Walker A.J., Kohler M.E., Venkateshwara V.R. (2015). 4-1BB costimulation ameliorates T cell exhaustion induced by tonic signaling of chimeric antigen receptors. Nat. Med..

[55] Ruella M., Kenderian S.S., Shestova O., Fraietta J.A., Qayyum S., Zhang Q., Maus M.V., Liu X., Nunez-Cru S., Klichinsky M. (2016). The addition of the BTK inhibitor ibrutinib to anti-CD19 chimeric antigen receptor T cells (CART19) improves responses against mantle cell lymphoma. Clin. Cancer Res..

[56] Fan F., Yoo H.J., Stock S., Wang L., Liu Y., Schubert M.L., Wang S., Neuber B., Hückelhoven-Krauss A., Gern U. (2021). Ibrutinib for improved chimeric antigen receptor T-cell production for chronic lymphocytic leukemia patients. Int. J. Cancer..

[57] Xu X., Sun Q., Liang X., Chen Z., Zhang X., Zhou X., Li M., Tu H., Liu Y., Tu S. (2019). Mechanisms of relapse after CD19 CAR T-cell therapy for acute lymphoblastic leukemia and its prevention and treatment strategies. Front. Immunol..

[58] Maude S.L., Laetsch T.W., Buechner J., Rives S., Boyer M., Bittencourt H., Bader P., Verneris M.R., Stefanski H.E., Myers G.D. (2018). Tisagenlecleucel in children and young adults with B-cell lymphoblastic leukemia. N. Engl. J. Med..

[59] Fischer J., Paret C., El Malki K., Alt F., Wingerter A., Neu M.A., Kron B., Russo A., Lehmann N., Roth L. (2017). CD19 isoforms enabling resistance to CART-19 immunotherapy are expressed in B-ALL patients at initial diagnosis. J. Immunother..

[60] Sotillo E., Barrett D.M., Black K.L., Bagashev A., Oldridge D., Wu G., Sussman R., Lanauze C., Ruella M., Gazzara M.R. (2015). Convergence of acquired mutations and alternative splicing of CD19 enables resistance to CART-19 immunotherapy. Cancer Discov..

[61] Orlando E.J., Han X., Tribouley C., Wood P.A., Leary R.J., Riester M., Levine J.E., Qayed M., Grupp S.A., Boyer M. (2018). Genetic mechanisms of target antigen loss in CAR19 therapy of acute lymphoblastic leukemia. Nat. Med..

[62] Pillai V., Muralidharan K., Meng W., Bagashev A., Oldridge D.A., Rosenthal J., Van Arnam J., Melenhorst J.J., Mohan D., DiNofia A.M. (2019). CAR T-cell therapy is effective for CD19-dim B-lymphoblastic leukemia but is impacted by prior blinatumomab therapy. Blood Adv..

[63] Fry T.J., Shah N.N., Orentas R.J., Stetler-Stevenson M., Yuan C.M., Ramakrishna S., Wolters P., Martin S., Delbrook C., Yates B. (2018). CD22-targeted CAR T cells induce remission in B-ALL that is naive or resistant to CD19-targeted CAR immunotherapy. Nat. Med..

[64] Ruella M., Maus M.V. (2016). Catch me if you can: Leukemia escape after CD19-directed T cell immunotherapies. Comput. Struct. Biotechnol. J..

[65] Rosenthal J., Naqvi A.S., Luo M., Wertheim G., Paessler M., Thomas-Tikhonenko A., Rheingold S.R., Pillai V. (2018). Heterogeneity of surface CD19 and CD22 expression in B lymphoblastic leukemia. Am. J. Hematol..

[66] Pan J., Tan Y., Deng B., Tong C., Hua L., Ling Z., Song W., Xu J., Duan J., Wang Z. (2020). Frequent occurrence of CD19-negative relapse after CD19 CAR T and consolidation therapy in 14 TP53-mutated r/r B-ALL children. Leukemia.

[67] Shah N.N., Stevenson M.S., Yuan C.M., Richards K., Delbrook C., Kreitman R.J., Pastan I., Wayne A.S. (2015). Characterization of CD22 expression in acute lymphoblastic leukemia. Pediatr. Blood Cancer.

[68] Wang Q.S., Wang Y., Lv H.Y., Han Q.W., Fan H., Guo B., Wang L.L., Han W.D. (2015). Treatment of CD33-directed chimeric antigen receptor-modified T cells in one patient with relapsed and refractory acute myeloid leukemia. Mol. Ther..

[69] Zhang W.-Y., Wang Y., Guo Y.-L., Dai H.-R., Yang Q.-M., Zhang Y.-J., Zhang Y., Chen M.-X., Wang C.-M., Feng K.-C. (2016). Treatment of CD20-directed chimeric antigen receptor-modified T cells in patients with relapsed or refractory B-cell non-Hodgkin lymphoma: An early phase IIa trial report. Signal Transduct. Targeted Ther..

[70] Lee D.W., Gardner R., Porter D.L., Louis C.U., Ahmed N., Jensen M., Grupp S.A., Mackall C.L. (2014). Current concepts in the diagnosis and management of cytokine release syndrome. Blood.

[71] Shah B.D., Bishop M.R., Oluwole O.O., Logan A., Baer M.R., Donnellan W.B., Carr-O’Dwyer K.M., Holmes H., Arellano M.L., Ghobadi A. (2019). End of phase I results of ZUMA-3, a phase 1/2 study of KTE-X19, anti-CD19 chimeric antigen receptor (CAR) T cell therapy, in adult patients (pts) with relapsed/refractory (R/R) acute lymphoblastic leukemia (ALL). J. Clin. Oncol..

[72] Davila M.L., Riviere I., Wang X., Bartido S., Park J., Curran K., Chung S.S., Stefanski J., Borquez-Ojeda O., Olszewska M. (2014). Efficacy and toxicity management of 19–28z CAR T cell therapy in B cell acute lymphoblastic leukemia. Sci. Transl. Med..

[73] Maude S.L., Frey N., Shaw P.A., Aplenc R., Barrett D.M., Bunin N.J., Chew A., Gonzalez V.E., Zheng Z., Lacey S.F. (2014). Chimeric antigen receptor T cells for sustained remissions in leukemia. N. Engl. J. Med..

[74] Lee D.W., Kochenderfer J.N., Stetler-Stevenson M., Cui Y.K., Delbrook C., Feldman S.A., Fry T.J., Orentas R., Sabatino M., Shah N.N. (2015). T cells expressing CD19 chimeric antigen receptors for acute lymphoblastic leukaemia in children and young adults: A phase 1 dose-escalation trial. Lancet.

[75] Neelapu S.S., Tummala S., Kebriaei P., Wierda W., Gutierrez C., Locke F.L., Komanduri K.V., Lin Y., Jain N., Daver N. (2018). Chimeric antigen receptor T-cell therapy - assessment and management of toxicities. Nat. Rev. Clin. Oncol..

[76] Mahadeo K.M., Khazal S.J., Abdel-Azim H., Fitzgerald J.C., Taraseviciute A., Bollard C.M., Tewari P., Duncan C., Traube C., McCall D. (2019). Management guidelines for paediatric patients receiving chimeric antigen receptor T cell therapy. Nat. Rev. Clin. Oncol..

[77] Maus M.V., Alexander S., Bishop M.R., Brudno J.N., Callahan C., Davila M.L., Diamonte C., Dietrich J., Fitzgerald J.C., Frigault M.J. (2020). Society for Immunotherapy of Cancer (SITC) clinical practice guideline on immune effector cell-related adverse events. J. Immunother. Cancer.

[78] Gust J., Hay K.A., Hanafi L.A., Li D., Myerson D., Gonzalez-Cuyar L.F., Yeung C., Liles W.C., Wurfel M., Lopez J.A. (2017). Endothelial activation and blood-brain barrier disruption in neurotoxicity after adoptive immunotherapy with CD19 CAR-T cells. Cancer Discov..

[79] Santomasso B.D., Park J.H., Salloum D., Riviere I., Flynn J., Mead E., Halton E., Wang X., Senechal B., Purdon T. (2018). Clinical and biological correlates of neurotoxicity associated with CAR T-cell therapy in patients with B-cell acute lymphoblastic leukemia. Cancer Discov..

[80] Lee D.W., Santomasso B.D., Locke F.L., Ghobadi A., Turtle C.J., Brudno J.N., Maus M.V., Park J.H., Mead E., Pavletic S. (2019). ASTCT consensus grading for cytokine release syndrome and neurologic toxicity associated with immune effector cells. Biol. Biol. Blood Marrow Transplant..

[81] Kranick S., Phan G., Kochenderfer J., Rosenberg S., Nath A. (2015). Aphasia as a complication of CD19-targeted chimeric antigen receptor immunotherapy (S52.006). Neurology.

[82] Cohen A.D., Garfall A.L., Stadtmauer E.A., Lacey S.F., Lancaster E., Vogl D.T., Dengel K., Ambrose D.E., Chen F., Plesa G. (2016). B-cell maturation antigen (BCMA)-specific chimeric antigen receptor T cells (CART-BCMA) for multiple myeloma (MM): Initial safety and efficacy from a phase I study. Blood.

[83] Shalabi H., Wolters P.L., Martin S., Delbrook C., Yates B., Lee D.W., Mackall C.L., Fry T.J., Shah N.N. A prospective evaluation of neurocognitive function and neurologic symptoms in pediatric and young adult patients with relapsed/refractory acute lymphoblastic leukemia (ALL) undergoing anti-CD22 chimeric antigen receptor therapy. Proceedings of the 58th ASH Annual Meeting and Exposition.

[84] Maude S.L., Teachey D.T., Porter D.L., Grupp S.A. (2015). CD19-targeted chimeric antigen receptor T-cell therapy for acute lymphoblastic leukemia. Blood.

[85] Harris K., LaBelle J.L., Bishop M.R. (2021). Current status of CAR T cell therapy for leukemias. Curr. Treat. Options. Oncol..

[86] Kochenderfer J.N., Wilson W.H., Janik J.E., Dudley M.E., Stetler-Stevenson M., Feldman S.A., Maric I., Raffeld M., Nathan D.A., Lanier B.J. (2010). Eradication of B-lineage cells and regression of lymphoma in a patient treated with autologous T cells genetically engineered to recognize CD19. Blood.

[87] Curran K.J., Pegram H.J., Brentjens R.J. (2012). Chimeric antigen receptors for T cell immunotherapy: Current understanding and future directions. J. Gene Med..

[88] Kochenderfer J.N., Dudley M.E., Feldman S.A., Wilson W.H., Spaner D.E., Maric I., Stetler-Stevenson M., Phan G.Q., Hughes M.S., Sherry R.M. (2012). B-cell depletion and remissions of malignancy along with cytokine-associated toxicity in a clinical trial of anti-CD19 chimeric-antigen-receptor-transduced T cells. Blood.

[89] Sandler R.D., Tattersall R.S., Schoemans H., Greco R., Badoglio M., Labopin M., Alexander T., Kirgizov K., Rovira M., Saif M. (2020). Diagnosis and management of secondary HLH/MAS following HSCT and CAR-T cell therapy in adults; A review of the literature and a survey of practice within EBMT centres on behalf of the Autoimmune Diseases Working Party (ADWP) and Transplant Complications Working Party (TCWP). Front. Immunol..

[90] (2023). ClinicalTrials.gov. Used Key Word: CAR-T Cells. https://clinicaltrials.gov/search?term=CAR%20T-cells%20&cond=Acute%20Lymphoblastic%20Leukemia.

[91] Pasquini M.C., Hu Z.H., Curran K., Laetsch T., Locke F., Rouce R., Pulsipher M.A., Phillips C.L., Keating A., Frigault M.J. (2020). Real-world evidence of tisagenlecleucel for pediatric acute lymphoblastic leukemia and non-Hodgkin lymphoma. Blood Adv..

[92] Anagnostou T., Riaz I.B., Hashmi S.K., Murad M.H., Kenderian S.S. (2020). Anti-CD19 chimeric antigen receptor T-cell therapy in acute lymphocytic leukaemia: A systematic review and meta-analysis. Lancet Haematol..

[93] Pan J., Niu Q., Deng B., Liu S., Wu T., Gao Z., Liu Z., Zhang Y., Qu X., Zhang Y. (2019). CD22 CAR T-cell therapy in refractory or relapsed B acute lymphoblastic leukemia. Leukemia.

[94] Dai H., Wu Z., Jia H., Tong C., Guo Y., Ti D., Han X., Liu Y., Zhang W., Wang C. (2020). Bispecific CAR-T cells targeting both CD19 and CD22 for therapy of adults with relapsed or refractory B cell acute lymphoblastic leukemia. J. Hematol. Oncol..

[95] Cordoba S., Onuoha S., Thomas S., Pignataro D.S., Hough R., Ghorashian S., Vora A., Bonney D., Veys P., Rao K. (2021). CAR T cells with dual targeting of CD19 and CD22 in pediatric and young adult patients with relapsed or refractory B cell acute lymphoblastic leukemia: A phase 1 trial. Nat. Med..

[96] Roddie C., Lekakis L.J., Marzolini M.A.V., Ramakrishnan A., Zhang Y., Hu Y., Peddareddigari V.G.R., Khokhar N., Chen R., Basilico S. (2023). Dual targeting of CD19 and CD22 with bicistronic CAR-T cells in patients with relapsed/refractory large B-cell lymphoma. Blood.

[97] Fergusson N.J., Adeel K., Kekre N., Atkins H., Hay K.A. (2023). A systematic review and meta-analysis of CD22 CAR T-cells alone or in combination with CD19 CAR T-cells. Front. Immunol..

[98] Fitzgerald J.C., Weiss S.L., Maude S.L., Barrett D.M., Lacey S.F., Melenhorst J.J., Shaw P., Berg R.A., June C.H., Porter D.L. (2017). Cytokine release syndrome after chimeric antigen receptor T cell therapy for acute lymphoblastic leukemia. Crit. Care. Med..

[99] Shah N.N., Lee D.W., Yates B., Yuan C.M., Shalabi H., Martin S., Wolters P.L., Steinberg S.M., Baker E.H., Delbrook C.P. (2021). Long-term follow-up of CD19-CAR T-cell therapy in children and young adults with B-ALL. J. Clin. Oncol..

[100] Frey N.V., Shaw P.A., Hexner E.O., Pequignot E., Gill S., Luger S.M., Mangan J.K., Loren A.W., Perl A.E., Maude S.L. (2020). Optimizing chimeric antigen receptor T-cell therapy for adults with acute lymphoblastic leukemia. J. Clin. Oncol..

[101] Gu R., Liu F., Zou D., Xu Y., Lu Y., Liu B., Liu W., Chen X., Liu K., Guo Y. (2020). Efficacy and safety of CD19 CAR T constructed with a new anti-CD19 chimeric antigen receptor in relapsed or refractory acute lymphoblastic leukemia. J. Hematol. Oncol..

[102] Grupp S.A., Maude S.L., Rives S., Baruchel A., Boyer M.W., Bittencourt H., Bader P., Büchner J., Laetsch T.W., Stefanski H. (2018). Updated analysis of the efficacy and safety of Tisagenlecleucel in pediatric and young adult patients with relapsed/refractory (r/r) acute lymphoblastic leukemia. Blood.

[103] Schultz L.M., Baggott C., Prabhu S., Pacenta H., Phillips C.L., Rossoff J., Stefanski H., Talano J.A., Moskop A., Margossian S.P. (2020). Disease burden impacts outcomes in pediatric and young adult B-cell acute lymphoblastic leukemia after commercial Tisagenlecleucel: Results from pediatric real-world CAR Consortium (PRWCC). Blood.

[104] Gardner R.A., Finney O., Annesley C., Brakke H., Summers C., Leger K., Bleakley M., Brown C., Mgebroff S., Kelly-Spratt K.S. (2017). Intent-to-treat leukemia remission by CD19 CAR T cells of defined formulation and dose in children and young adults. Blood.

[105] Curran K.J., Margossian S., Kernan N.A., Silverman L.B., Williams D.A., Shukla N.N., Kobos R., Forlenza C.J., Steinherz P., Prockop S. (2019). Toxicity and response following CD19-specific CAR T cells in pediatric/young adult relapsed/refractory B-ALL. Blood.

[106] Turtle C.J., Hanafi L.-A., Berger C., Gooley T.A., Cherian S., Hudecek M., Sommermeyer D., Melville K., Pender B., Budiarto T.M. (2016). CD19 CAR–T cells of defined CD4+: CD8+ composition in adult B cell ALL patients. J. Clin. Investig..

[107] Hay K.A., Gauthier J., Hirayama A.V., Voutsinas J.M., Wu Q., Li D., Gooley T.A., Cherian S., Chen X., Pender B.S. (2019). Factors associated with durable EFS in adult B-cell ALL patients achieving MRD-negative CR after CD19 CAR T-cell therapy. Blood.

[108] Ghorashian S., Kramer A.M., Onuoha S., Wright G., Bartram J., Richardson R., Albon S.J., Casanovas-Company J., Castro F., Popova B. (2019). Enhanced CAR T cell expansion and prolonged persistence in pediatric patients with ALL treated with a low-affinity CD19 CAR. Nat. Med..

[109] Ghorashian S., Kramer A.M., Onuoha S., Wright G., Bartram J., Richardson R., Albon S.J., Casanovas-Company J., Castro F., Popova B. (2019). Therapy of paediatric B-ALL with a fast off rate CD19 CAR leads to enhanced expansion and prolonged CAR T cell persistence in patients with low bone marrow tumour burden, and is associated with a favourable toxicity profile. Blood.

[110] Yang J., Jiang P., Zhang X., Zhu X., Dong Q., He J., Lin N., Wang Z., Cai S., Ye X. (2019). Anti-CD19/CD22 dual CAR-T therapy for refractory and relapsed B-cell acute lymphoblastic leukemia. Blood.

[111] Gardner R.A., Annesley C., Wilson A., Summers C., Narayanaswamy P., Wu V., Huang W., Johnson A., Li A., Schultz K.R. (2020). Efficacy of SCRI-CAR19x22 T cell product in B-ALL and persistence of anti-CD22 activity. J. Clin. Oncol..

[112] Zhang X., Lu X.A., Yang J., Zhang G., Li J., Song L., Su Y., Shi Y., Zhang M., He J. (2020). Efficacy and safety of anti-CD19 CAR T-cell therapy in 110 patients with B-cell acute lymphoblastic leukemia with high-risk features. Blood Adv..

[113] Shah B.D., Ghobadi A., Oluwole O.O., Logan A.C., Boissel N., Cassaday R.D., Leguay T., Bishop M.R., Topp M.S., Tzachanis D. (2021). KTE-X19 for relapsed or refractory adult B-cell acute lymphoblastic leukaemia: Phase 2 results of the single-arm, open-label, multicentre ZUMA-3 study. Lancet.

[114] Wayne A.S., Huynh V., Hijiya N., Rouce R.H., Brown P.A., Krueger J., Kitko C.L., Ziga E.D., Hermiston M.L., Richards M.K. (2023). Three-year results from phase I of ZUMA-4: KTE-X19 in pediatric relapsed/refractory acute lymphoblastic leukemia. Haematologica.

[115] Depil S., Duchateau P., Grupp S.A., Mufti G., Poirot L. (2020). ‘Off-the-shelf’ allogeneic CAR T cells: Development and challenges. Nat. Rev. Drug. Discov..

[116] Papathanasiou M.M., Stamatis C., Lakelin M., Farid S., Titchener-Hooker N., Shah N. (2020). Autologous CAR T-cell therapies supply chain: Challenges and opportunities?. Cancer Gene Ther..

[117] Das R.K., Vernau L., Grupp S.A., Barrett D.M. (2019). Naïve T-cell deficits at diagnosis and after chemotherapy impair cell therapy potential in pediatric cancers. Cancer Discov..

[118] Tang T.C.Y., Xu N., Nordon R., Haber M., Micklethwaite K., Dolnikov A. (2022). Donor T cells for CAR T cell therapy. Biomark. Res..

[119] Shah N.N., Fry T.J. (2019). Mechanisms of resistance to CAR T cell therapy. Nat. Rev. Clin. Oncol..

[120] Zhang C., He J., Liu L., Wang J., Wang S., Liu L., Ge J., Gao L., Gao L., Kong P. (2022). Novel CD19 chimeric antigen receptor T cells manufactured next-day for acute lymphoblastic leukemia. Blood Cancer J..

[121] Mo F., Srinivasan M., Heslop H.E., Brenner M.K., Mamonkin M. (2019). Rejection-resistant off-the-shelf T cells for adoptive cell therapy. Biol. Blood Marrow Transplant..

[122] Poirot L., Philip B., Schiffer-Mannioui C., Le Clerre D., Chion-Sotinel I., Derniame S., Potrel P., Bas C., Lemaire L., Galetto R. (2015). Multiplex genome-edited T-cell manufacturing platform for “off-the-shelf” adoptive T-cell immunotherapies. Cancer Res..

[123] Ren J., Liu X., Fang C., Jiang S., June C.H., Zhao Y. (2017). Multiplex genome editing to generate universal CAR T cells resistant to PD1 inhibition. Clin. Cancer Res..

[124] Cooper M.L., Choi J., Staser K., Ritchey J.K., Devenport J.M., Eckardt K., Rettig M.P., Wang B., Eissenberg L.G., Ghobadi A. (2018). An “off-the-shelf” fratricide-resistant CAR-T for the treatment of T cell hematologic malignancies. Leukemia.

[125] Qasim W., Zhan H., Samarasinghe S., Adams S., Amrolia P., Stafford S., Butler K., Rivat C., Wright G., Somana K. (2017). Molecular remission of infant B-ALL after infusion of universal TALEN gene-edited CAR T cells. Sci. Transl. Med..

[126] Benjamin R., Graham C., Yallop D., Jozwik A., Mirci-Danicar O.C., Lucchini G., Pinner D., Jain N., Kantarjian H., Boissel N. (2020). Genome-edited, donor-derived allogeneic anti-CD19 chimeric antigen receptor T cells in paediatric and adult B-cell acute lymphoblastic leukaemia: Results of two phase 1 studies. Lancet..

[127] Mo F., Watanabe N., McKenna M.K., Hicks M.J., Srinivasan M., Gomes-Silva D., Atilla E., Smith T., Ataca Atilla P., Ma R. (2021). Engineered off-the-shelf therapeutic T cells resist host immune rejection. Nat. Biotechnol..

[128] Torikai H., Reik A., Liu P.Q., Zhou Y., Zhang L., Maiti S., Huls H., Miller J.C., Kebriaei P., Rabinovitch B. (2012). A foundation for universal T-cell based immunotherapy: T cells engineered to express a CD19-specific chimeric-antigen-receptor and eliminate expression of endogenous TCR. Blood.

[129] Cruz C.R.Y., Micklethwaite K.P., Savoldo B., Ramos C.A., Lam S., Ku S., Diouf O., Liu E., Barrett A.J., Ito S. (2013). Infusion of donor-derived CD19-redirected virus-specific T cells for B-cell malignancies relapsed after allogeneic stem cell transplant: A phase 1 study. Blood.

[130] Leen A.M., Bollard C.M., Mendizabal A.M., Shpall E.J., Szabolcs P., Antin J.H., Kapoor N., Pai S.Y., Rowley S.D., Kebriaei P. (2013). Multicenter study of banked third-party virus-specific T cells to treat severe viral infections after hematopoietic stem cell transplantation. Blood.

[131] Tzannou I., Papadopoulou A., Naik S., Leung K., Martinez C.A., Ramos C.A., Carrum G., Sasa G., Lulla P., Watanabe A. (2017). Off-the-shelf virus-specific T cells to treat BK virus, human herpesvirus 6, cytomegalovirus, Epstein-Barr virus, and adenovirus infections after allogeneic hematopoietic stem-cell transplantation. J. Clin. Oncol..

[132] Eyquem J., Mansilla-Soto J., Giavridis T., van der Stegen S.J., Hamieh M., Cunanan K.M., Odak A., Gönen M., Sadelain M. (2017). Targeting a CAR to the TRAC locus with CRISPR/Cas9 enhances tumour rejection. Nature.

[133] Ottaviano G., Georgiadis C., Gkazi S.A., Syed F., Zhan H., Etuk A., Preece R., Chu J., Kubat A., Adams S. (2022). Phase 1 clinical trial of CRISPR-engineered CAR19 universal T cells for treatment of children with refractory B cell leukemia. Sci. Transl. Med..

[134] Saura-Esteller J., de Jong M., King L.A., Ensing E., Winograd B., de Gruijl T.D., Parren P.W.H.I., van der Vliet H.J. (2022). Gamma delta T-cell based cancer immunotherapy: Past-present-future. Front. Immunol..

[135] Mehta R.S., Rezvani K. (2018). Chimeric Antigen Receptor Expressing Natural Killer Cells for the Immunotherapy of Cancer. Front. Immunol..

[136] Liu E., Marin D., Banerjee P., Macapinlac H.A., Thompson P., Basar R., Nassif Kerbauy L., Overman B., Thall P., Kaplan M. (2020). Use of CAR-transduced natural killer cells in CD19-Positive lymphoid tumors. N. Engl. J. Med..

[137] (2023). ClinicalTrials.gov. Used Key Word: CAR-NK. https://clinicaltrials.gov/search?term=CAR-NK.

[138] Klichinsky M., Ruella M., Shestova O., Lu X.M., Best A., Zeeman M., Schmierer M., Gabrusiewicz K., Anderson N.R., Petty N.E. (2020). Human chimeric antigen receptor macrophages for cancer immunotherapy. Nat. Biotechnol..

[139] Chen Y., Yu Z., Tan X., Jiang H., Xu Z., Fang Y., Han D., Hong W., Wei W., Tu J. (2021). CAR-macrophage: A new immunotherapy candidate against solid tumors. Biomed. Pharmacother..

[140] (2023). ClinicalTrials.gov. Used Key Word: CAR-Macrophages. https://clinicaltrials.gov/search?term=CAR-Macrophages.

[141] Zhang T., Wang T., You F., Li Z., Chen D., Zhang K., Tian S., Sheng B., Wu H., Jiang L. (2022). Nanobody-based anti-CD22-chimeric antigen receptor T cell immunotherapy exhibits improved remission against B-cell acute lymphoblastic leukemia. Transpl. Immunol..

[142] Wei J., Han X., Bo J., Han W. (2019). Target selection for CAR-T therapy. J. Hematol. Oncol..

[143] Martyniszyn A., Krahl A.C., André M.C., Hombach A.A., Abken H. (2017). CD20-CD19 Bispecific CAR T Cells for the Treatment of B-Cell Malignancies. Hum. Gene. Ther..

[144] Schultz L.M., Muffly L.S., Spiegel J.Y., Ramakrishna S., Hossain N., Baggott C., Sahaf B., Patel S., Craig J., Yoon J. Phase I trial using CD19/CD22 bispecific CAR T cells in pediatric and adult acute lymphoblastic leukemia (ALL). Proceedings of the 61nd ASH Annual Meeting and Exposition.

[145] Yang J., Jiang P., Zhang X., Li J., Wu Y., Xu L., Su Y., Hu X., Zhao X., Dong Q. Successful 24-hours manufacture of anti-CD19/CD22 dual chimeric antigen receptor (CAR) T cell therapy for B-cell acute lymphoblastic leukemia (B-ALL) clinically relevant abstract. Proceedings of the 62nd ASH Annual Meeting and Exposition.

[146] Yan L.E., Zhang H., Wada M., Fang L., Feng J., Zhang W., Chen Q., Cao Y., Pinz K.G., Chen K.H. (2020). Targeting two antigens associated with B-ALL with CD19-CD123 compound Car T cell therapy. Stem. Cell. Rev. Rep..

[147] Wang X., Dong Z., Awuah D., Chang W.C., Cheng W.A., Vyas V., Cha S.C., Anderson A.J., Zhang T., Wang Z. (2022). CD19/BAFF-R dual-targeted CAR T cells for the treatment of mixed antigen-negative variants of acute lymphoblastic leukemia. Leukemia.

[148] Fousek K., Watanabe J., George A., An X., Samaha H.S., Navai S.A., Byrd T.T., Jang A., Kim H., Sujith J. (2018). Targeting CD19-negative relapsed B-acute lymphoblastic leukemia using trivalent CAR T cells. J. Clin. Oncol..

[149] Yan N., Wang N., Wang G., Huang L., Li C., Wang D., Wang J., Huang L., Meng F., Wei J. (2022). CAR19/22 T cell cocktail therapy for B-ALL relapsed after allogeneic hematopoietic stem cell transplantation. Cytotherapy.

[150] Teachey D.T., O’Connor D. (2020). How I treat newly diagnosed T-cell acute lymphoblastic leukemia and T-cell lymphoblastic lymphoma in children. Blood..

[151] Chen K.H., Wada M., Pinz K.G., Liu H., Lin K.W., Jares A., Firor A.E., Shuai X., Salman H., Golightly M. (2017). Preclinical targeting of aggressive T-cell malignancies using anti-CD5 chimeric antigen receptor. Leukemia.

[152] Png Y.T., Vinanica N., Kamiya T., Shimasaki N., Coustan-Smith E., Campana D. (2017). Blockade of CD7 expression in T cells for effective chimeric antigen receptor targeting of T-cell malignancies. Blood Adv..

[153] Huang J., Alexey S., Li J., Jones T., Grande G., Douthit L., Xie J., Chen D., Wu X., Michael M. (2019). Unique CDR3 epitope targeting by CAR-T cells is a viable approach for treating T-cell malignancies. Leukemia.

[154] Voynova E., Hawk N., Flomerfelt F.A., Telford W.G., Gress R.E., Kanakry J.A., Kovalovsky D. (2022). Increased activity of a NK-specific CAR-NK framework targeting CD3 and CD5 for T-cell leukemias. Cancers.

[155] You F., Wang Y., Jiang L., Zhu X., Chen D., Yuan L., An G., Meng H., Yang L. (2019). A novel CD7 chimeric antigen receptor-modified NK-92MI cell line targeting T-cell acute lymphoblastic leukemia. Am. J. Cancer Res..

[156] Ren A., Tong X., Xu N., Zhang T., Zhou F., Zhu H. (2023). CAR T-cell immunotherapy treating T-ALL: Challenges and opportunities. Vaccines.

[157] Zheng N.S., Zhao X.Y., Wei D., Miao J.L., Liu Z.K., Yong Y.L., Zhang R.Y., Guo Y.X., He L., Wang B. (2022). CD147-specific chimeric antigen receptor T cells effectively inhibit T cell acute lymphoblastic leukemia. Cancer Lett..

[158] Dai H.P., Cui W., Cui Q.Y., Zhu W.J., Meng H.M., Zhu M.Q., Zhu X.M., Yang L., Wu D.P., Tang X.W. (2022). Haploidentical CD7 CAR T-cells induced remission in a patient with TP53 mutated relapsed and refractory early T-cell precursor lymphoblastic leukemia/lymphoma. Biomark. Res..

